# *Taenia solium* Infection in Peru: A Collaboration between Peace Corps Volunteers and Researchers in a Community Based Study

**DOI:** 10.1371/journal.pone.0113239

**Published:** 2014-12-03

**Authors:** Nathaniel S. Watts, Monica Pajuelo, Taryn Clark, Maria-Cristina I. Loader, Manuela R. Verastegui, Charles Sterling, Jon S. Friedland, Hector H. Garcia, Robert H. Gilman, for the Cysticercosis Working Group in Peru

**Affiliations:** 1 Department of Epidemiology, Boston University School of Public Health, Boston, United States of America; 2 Unit of Bioinformatics and Molecular Biology, School of Sciences and Philosophy, Universidad Peruana Cayetano Heredia, Lima, Peru; 3 Weill Cornell Medical College, New York, United States of America; 4 Infectious Diseases & Immunity and the Wellcome Trust-Imperial Centre for Global Health, Imperial College London, London, England, United Kingdom; 5 Department of Microbiology, School of Sciences and Philosophy, Universidad Peruana Cayetano Heredia, Lima, Peru; 6 School of Animal and Comparative Biomedical Sciences, University of Arizona, Tucson, United States of America; 7 Asociación Benéfica Proyectos en Informática, Salud, Medicina y Agricultura (AB PRISMA), Lima, Peru; 8 Department of International Health, Bloomberg School of Public Health, Johns Hopkins University, Baltimore, United States of America; Kliniken der Stadt Köln gGmbH, Germany

## Abstract

**Background:**

Neurocysticercosis is a leading cause of seizures and epilepsy in most of the world, and it occurs when *Taenia solium* larval cysts infect the central nervous system. *T. solium* tapeworm infection is endemic in much of Peru, but there are scarce data on the prevalence in many rural highland communities where it is likely to be hyper-endemic. Peace Corps Volunteers live and work in these communities; however, to our knowledge, they have not been used to facilitate public health research.

**Materials and Methods:**

We utilized Peace Corps Volunteers to estimate the prevalence of *T. solium* tapeworm infection in seven rural communities in northern Peru. A convenience non-random sampling frame was used. Peace Corps Volunteers facilitated the collection of stool samples (N = 2,328), which were analyzed by sedimentation and microscopy. Niclosamide treatment and purgation preceded species identification, which was done by PCR-REA.

**Results:**

*Taenia* sp. egg-positive stool samples were found in three of the seven communities we surveyed. The overall prevalence of *Taenia* sp. egg positivity was 2.1% (49/2,328) (95% CI = 1.6–2.8%) with prevalence up to 4.3% (42/977) (95% CI = 3.1–5.8%) by community. All 34 of the specimens tested by PCR-REA were *T. solium*. The overall prevalence of *T. solium* tapeworm infection was 1.5% (34/2,328) (95% CI = 1.0–2.0%). Prevalence up to 2.9% (28/977) (95% CI = 1.9–4.1%) by community was observed.

**Conclusion/Significance:**

This study recorded high *T. solium* tapeworm prevalence, and identified hyper-endemic rural communities. It demonstrates that synergy between researchers and Peace Corps Volunteers can be an effective means to conducting large-scale, community-based studies in remote areas of Peru.

## Introduction

*Taenia solium (T. solium)* neurocysticercosis (NCC) is the leading cause of adult-acquired epilepsy worldwide, and an increasingly important public health problem in developed countries with migrant populations. [1], [2] Cysticercosis has been shown to cluster around *T. solium* tapeworm carriers, [3] as *T. solium* tapeworm infection not only increases carriers’ risk for NCC, but also places other household members at substantially elevated risks. [4], [5] *T. solium* tapeworm infection may be underreported and difficult to detect in rural, Andean communities due to a lack of awareness and diagnostic facilities. [4].

Estimating the prevalence of *T. solium* infection in such communities is necessary to allow researchers and public health workers to address this health problem. Conducting epidemiologic research in remote communities can be costly, time-consuming, and difficult due to their geographic locations, linguistic and cultural barriers, as well as the need to establish a working relationship with community leaders and government. Therefore, our group utilized an existing network of Peace Corps Volunteers (PCVs) who were integrated into their communities to perform epidemiologic screening and surveillance. Our consortium aimed to perform a large-scale, cross-sectional epidemiologic study to examine the feasibility of bringing together PCVs and researchers to perform a *T. solium* tapeworm prevalence study in multiple rural regions of northern Peru. To our knowledge, collaboration between a group of university investigators and PCVs to perform local surveillance of *T. solium* tapeworm infection is unprecedented.

## Materials and Methods

### Study area and population

**Figure 1:**
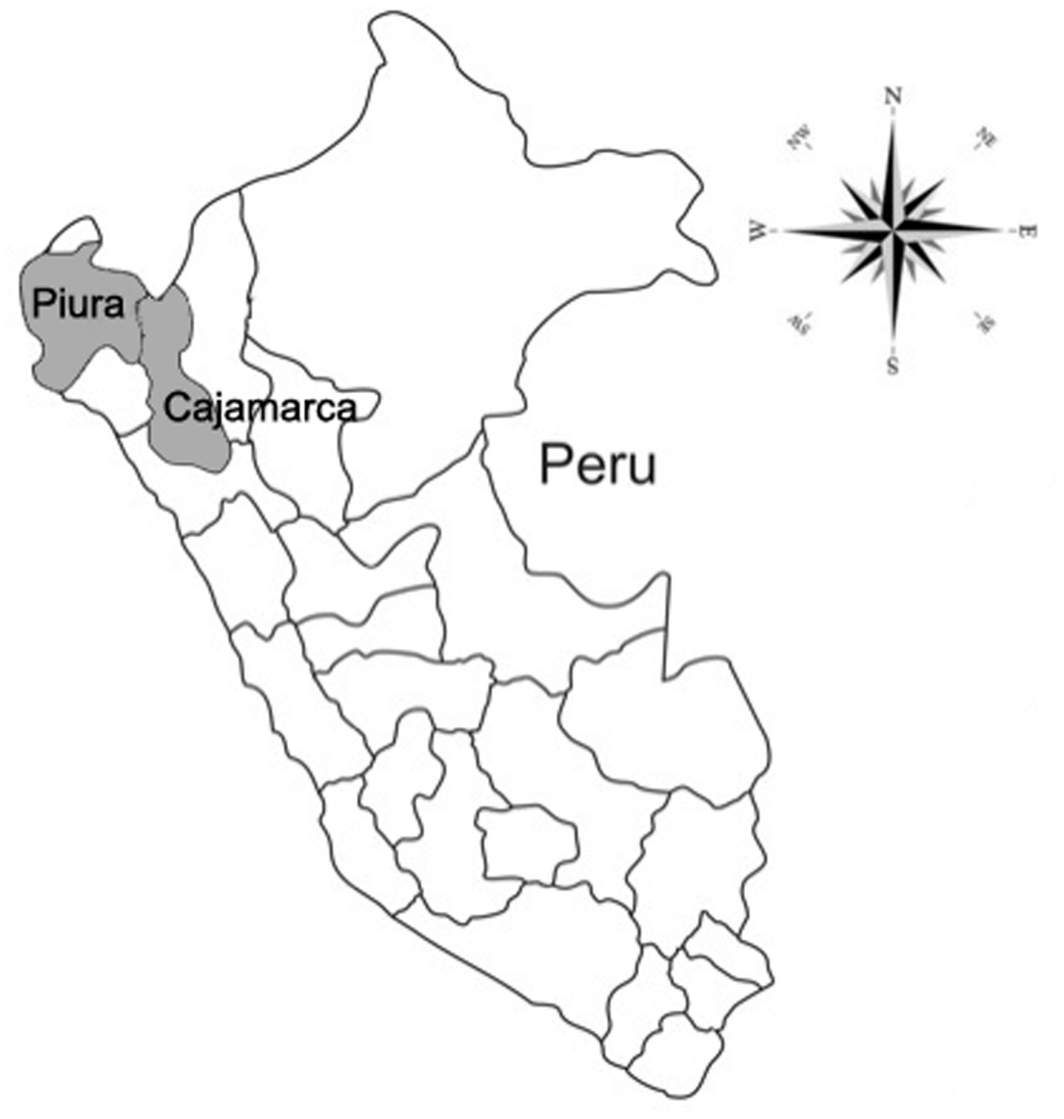
Map of Peru Showing Piura and Cajamarca.

The study was conducted in seven communities in the northern departments of Piura and Cajamarca (Fig. 1) following PCV reports of free-roaming pigs and inadequate access to latrines. All communities are rural and most inhabitants do not have access to reliable potable water for drinking nor adequate sanitation systems. Study sites ranged in altitude between 236–2,667 meters above sea level. The climate is hot at lower elevations, but becomes increasingly temperate with altitude. May through December is typically dry, with heavy rainfall from January to April. Agriculture is the main economic activity in these regions where villagers frequently raise pigs for consumption and sale. Peace Corps Volunteers suggested communities for the study based on the guiding principles for selection: the observed presence of pigs (usually free-roaming) and an inadequate access to latrines. Persons were included in the study if they resided in these communities, and persons were excluded if they resided in a community where a mass-deworming program was in effect. The study was conducted in the following communities (population data obtained from the last available nation census, 2007): Joras (Ayabaca district, 38,730 inhabitants), Oxahuay (Sicchez district, 2,274 inhabitants), Pampa Elera Baja (Las Lomas district, 26,896 inhabitants), Baños del Inca (Baños del Inca district, 36,800 inhabitants), Chalamarca (Chalamarca district, 10,530 inhabitants), Iraca Grande (Chota district, 45,555 inhabitants), and Conchan (Conchan district, 6,449 inhabitants).

### Study design

**Figure 2:**
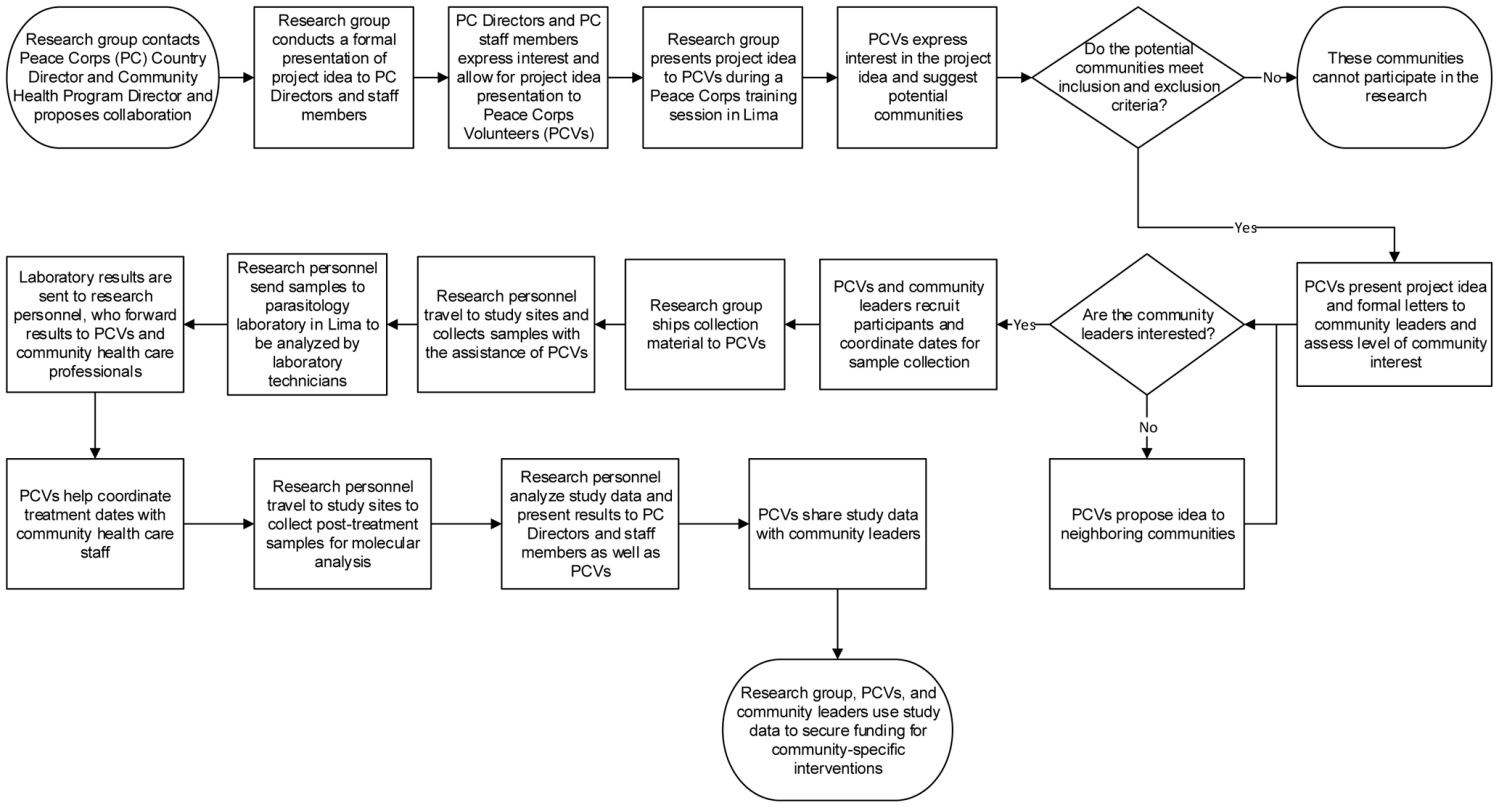
Process Map Demonstrating Key Steps in Collaboration.

A community-based, cross-sectional prevalence study was performed using a convenience non-random sampling frame. The Cysticercosis Working Group (CWG) in Peru contacted the Country Director and Community Health Program Director at the Peace Corps office in Lima, Peru, and proposed collaboration. Researchers presented the study idea to Peace Corps staff and to 23 Health Program PCVs attending an in-service training session in Lima. Formal letters in Spanish explaining the research objective were sent to each PCV who suggested an eligible community. Peace Corps Volunteers presented these letters to their communities’ health posts, municipalities, schools, and community leaders. Peace Corps Volunteers worked in conjunction with leaders from these institutions to recruit participants. To maximize participation, all community residents were eligible to participate in this study. Figure 2 provides a process map detailing collaboration.

### Sample collection and analysis

Single stool specimens were collected from participants and analyzed for the presence of eggs, cysts, or parasite material using spontaneous sedimentation in tube technique (SSTT) and microscopy. [6] Each consenting resident was provided with a 500-ml plastic container with lid for collection of a whole, individual stool sample. Research personnel, with the assistance of PCVs, collected stool samples along with participant age and sex information. A 10cc fecal sample was placed in 40 cc of 5% of formol-Phosphate Buffered Saline, pH 7.2 (PBS) in a sealed propylene tube at room temperature. Fecal samples were transported at room temperature to the parasitology laboratory at Universidad Peruana Cayetano Heredia (UPCH) in Lima for analysis. Participants who were *Taenia* sp. egg-positive were invited to their community health clinic, where a local physician provided treatment. Treatment consisted of niclosamide and an electrolyte-polyethyleneglycol solution (EPS) purge (NuLYTELY, Asofarma, Argentina). [7] Adults were treated with 2 g of niclosamide and 2 L of EPS, which was given 2 h before and after treatment. Children>34 kg were given 1.5 g of niclosamide and 1 L of EPS. Children 11–34 kg were given 1 g of niclosamide and 25–40 mL/kg per h of EPS, divided into 8 doses. All participants were provided with detailed information on the risks associated with *T. solium* tapeworm infection and how to avoid repeat infection. Participants who did not expel the parasite were offered retreatment. All stools produced 24 hours post-treatment were examined for scoleces and proglottids, which were preserved in 95% alcohol for species identification by molecular analysis. Tapeworm material collected post-treatment was stored in 25% glycerol supplemented with penicillin (1000 Ul/ml) and gentamycin (100 Ug/ml) at 4°C until use. For *Taenia* species identification, DNA was isolated from proglottids expelled post-niclosamide treatment by phenol-chloroform, and a polymerase chain reaction (PCR) followed by restriction enzyme analysis (REA) was performed. [8] Briefly, PCR was performed in 25 µL master mix consisting in 1×PCR buffer (Gibco, Life Technologies) containing 2.5 mM MgCl_2_, 0.2 mM (each) dATP, dGTP, dCTP, and dTTP, 0.5 µM each primer, and 1 U of *Taq* polymerase. PCR was carried out in a thermocycler MJ Research MiniCycler PTC-150 with hot cover with the following temperature profile: 5 min at 94°C followed by 30 cycles consisting of 94°C for 1 min (denaturation), 56°C for 1 min (annealing), and 72°C for 2 min (elongation). Seventeen microliters of the PCR product was digested in 1 x enzyme buffer, 1 U of enzyme (*Alu*I, *Dde*I, or *Mbo*I). The reaction mixture was incubated for 3 h at 37°C. Finally, the products were separated by electrophoresis on a 1.0% agarose gel containing 0.5 µg of ethidium bromide/ml to evaluate *T. solium* pattern.

### Statistical Analysis

Prevalence (and 95% confidence intervals (CI)) of *Taenia* sp. and other intestinal parasitic infections was calculated as the number of stool samples that tested positive for eggs, or parasite material divided by the number of participants who contributed samples. Prevalence of *T. solium* tapeworm infection was calculated as the number of PCR-positive reaction divided by the number of participants who contributed samples. Pearson’s chi-square test was used to explore associations between age and sex and *T. solium* infection status, and a two proportions z-test was used to compare the proportion of parasitic infections between Cajamarca and Piura; p<0.05 was taken as statistically significant. All analyses were performed using Excel (Microsoft Office 2010) and STATA 10.1 (College Station, TX, USA).

### Ethics Statement

We obtained Institutional Review Board (IRB) approval from Universidad Peruana Cayetano Heredia (Protocol IRB-61133) in Lima, Peru, as well as *Asociación Benéfica Proyectos en Informática, Salud, Medicina y Agricultura (PRISMA)*, a non-governmental organization based in Lima, Peru. Appropriate informed consent procedures were followed. Adults (18 years of age and older) provided written informed consent. For subjects less than 18 years of age, written informed assent was obtained. Parents or guardians also provided written informed consent for minors.

## Results

### Study population

Eleven PCVs suggested a total of 10 potential communities for the study. One community was excluded because an existing cysticercosis elimination program was in effect, and community leaders from two communities declined to participate. A total of 2,328 participants from seven communities provided a stool sample. 2,169 (93.2%) participants provided information on sex and 2,136 (91.8%) provided information on age. 1,990 (85.5%) participants provided information on age and sex. 973 (44.9%) participants were male and 1,196 (55.1%) participants were female. The median age was 18 years IQR (9–40 years). Participants ranged in age from 1<year to 98 years. 1,485 (63.8%) participants were from Piura and 843 (36.2%) were from Cajamarca. The number of study participants in each community relative to the total inhabitants in the community is as follows: Joras (977/1,363), Oxahuay (88/640), Pampa Elera Baja (420/691), Baños del Inca (33/9,442), Chalamarca (550/776), Iraca Grande (96/957), and Conchan (164/419). Study population characteristics are presented in Table 1.

**Table 1:** Study Population Characteristics.

Department	Piura			Cajamarca				
Community	Joras	Oxahuay	Pampa EleraBaja	Baños delInca	Chalamarca	IracaGrande	Conchan	Total
No. of residents in community^a^	1,363	640	691	9,442	776	957	419	14,101
Sample size (n)	977	88	420	33	550	96	164	N = 2,328
Female, n (%)	432 (52.3%)	44 (50%)	232 (55.4%)	25 (75.8%)	301 (55.4%)	65 (67.7%)	97 (59.2%)	1,196 (55.1%)
Male, n (%)	394 (47.7%)	44 (50%)	187 (44.6%)	8 (24.2%)	242 (44.6%)	31 (32.3%)	67 (40.9%)	973 (44.9%)
No. who providedage, n (%)	844 (86.4%)	82 (93.2%)	420 (100%)	33 (100%)	504 (91.6%)	89 (92.7%)	164 (100%)	2,136 (91.8%)
No. who providedage, sex, n (%)	699 (71.0%)	82 (93.2%)	419 (99.8%)	33 (100%)	504 (91.6%)	89 (92.7%)	164 (100%)	1,990 (85.5%)
Median age (IQR)	16 (9–45)	15 (5–33)	15 (7–35)	14 (10–37)	25 (10–41)	22 (8–36)	17 (8–39)	18 (9–40)
Min, Max	<1, 90	1, 66	<1, 85	6, 54	<1, 98	<1, 91	1, 87	<1, 98

a Data obtained from 2007 national census.

### Prevalence of Taenia sp. egg positivity and T. solium tapeworm infection

In the total study population, the overall prevalence of *Taenia* sp. infection was 2.1% (49/2,328) (95% CI = 1.6–2.8%), and the overall prevalence of *T. solium* infection was 1.5% (34/2,328) (95% CI = 1.0–2.0%) (Table 2). Notably, no *Taenia* sp. egg-positive stool samples were found in Cajamarca. In Piura, the overall prevalence of *Taenia* sp. infection was 3.3% (49/1,458) (95% CI = 0.2–4.3%), reaching up to 4.3% (42/977) (95% CI = 3.1–5.8%) by community, and the overall prevalence of *T. solium* infection was 2.3% (34/1,485) (95% CI = 1.6–3.2%), reaching up to 2.9% (28/977) (95% CI = 1.9–4.1%) by community. Of the 49 *Taenia* sp. egg-positive cases, 39 were treated; 10 did not present for treatment for reasons unknown. From the treated group, 34 expelled tapeworm proglottids, which were all confirmed to be *T. solium*. Because we could not identify the species of *Taenia* in 15 egg-positive participants, *T. solium* tapeworm infection and *Taenia* sp. tapeworm infection prevalence are presented separately (Table 2). In Piura, *T. solium* was more prevalent in adults (18 years or older) (2.66%, 17/638) than children (1.84%, 13/708), although not statistically significant (*χ* ^2^ = 1.06, p = 0.30). *T. solium* tapeworm infection was also more prevalent in men (2.56%, 16/625) than women (2.54%,18/708), although this difference was not statistically significant (*χ* ^2^ = 0.0004, p = 0.98).

**Table 2:** Prevalence of *Taenia solium* and *Taenia* sp. Tapeworm Infection.

Department	Piura									
Community	Joras		Oxahuay		Las Lomas		Total in Piura		Total in study population	
	n = 977	95% CI	n = 88	95% CI	n = 420	95% CI	n = 1,485	95% CI	N = 2,328	95% CI
*Taenia solium*	2.9%	(1.9–4.1%)	2.3%	(0.2–8.0%)	1.0%	(0.3–2.4%)	2.3%	(1.6–3.2%)	1.5%	(1.0–2.0%)
*Taenia* sp.	4.3%	(3.1–5.8%)	3.4%	(0.7–9.6%)	1.0%	(0.3–2.4%)	3.3%	(0.2–4.3%)	2.1%	(1.6–2.8%)

### Prevalence of other parasites

Other intestinal parasites found in stool samples were *Ascaris lumbricoides (A. lumbricoides)*, hookworms, *Enterobius vermicularis, Giardia lamblia (G. lamblia), Strongyloides stercoralis*, and *Trichuris trichiura (T. trichiura)* (Tables 3 and 4). Prevalence of parasitic infection was found to be lower in Cajamarca than in Piura. This difference was statistically significant for *A. lumbricoides* (χ^2^ = 22.54, p<0.01), *G. lamblia* (χ^2^ = 23.63, p<0.01), and *T. trichiura* (χ^2^ = 5.84, p = 0.016). The most prevalent parasite in Cajamarca and Piura was *G. lamblia*.

**Table 3:** Prevalence of other Parasites by Community in Piura.

Department	Piura							
Community	Joras		Oxahuay		Las Lomas		Total	
	n = 977	95% CI	n = 88	95% CI	n = 420	95% CI	n = 1,485	95% CI
*Ascaris lumbricoides*	6.7%	(5.1–8.3%)	6.8%	(2.5–14.3%)	0.7%	(0.1–2.1%)	4.9%	(3.9–6.1%)
Hookworms	0		0		0.7%	(0.1–2.1%)	0.02%	(0.0–0.6%)
*Enterobius vermicularis*	1.1%	(0.5–1.9%)	0		0		0.7%	(0.3–1.2%)
*Giardia lamblia*	8.1%	(6.3–9.7%)	10.2%	(4.8–18.5%)	16.7%	(10.6–13.2%)	10.5%	(9.0–12.2%)
*Strongyloides stercoralis*	0.5%	(0.1–1.2%)	0		1.0%	(0.3–2.4%)	0.6%	(0.2–1.1%)
*Trichuris trichiura*	2.3%	(1.4–3.4%)	3.4%	(0.7–9.6%)	0.2%	(0.0–1.3%)	1.8%	(0.1–2.6%)

**Table 4:** Prevalence of other Parasites by Community in Cajamarca.

Department	Cajamarca									
Community	Baños del Inca		Chalamarca		Chota		Conchan		Total	
	n = 33	95%CI	n = 550	95%CI	n = 96	95%CI	n = 164	95%CI	n = 843	95%CI
*Ascaris* *lumbricoides*	3.0%	(0.0–15.8%)	2.2%	(1.1–3.8%)	4.2%	(1.1–10.3%)	1.8%	(0.3–5.3%)	2.4%	(1.5–3.6%)
Hookworms	0		0.2%	(0.0–5.7%)	1.0%	(0.0–5.7%)	0		0.2%	(0.0–0.9%)
*Enterobius* *vermicularis*	3.0%	(0.0–15.8%)	0.2%	(0.0–5.7%)	0		0		0.2%	(0.0–0.9%)
*Giardia lamblia*	3.0%	(0.0–15.8%)	4.6%	(2.9–6.6%)	2.1%	(0.2–7.3%)	6.7%	(3.4–11.7%)	4.7%	(3.3–6.3%)
*Strongyloides* *stercoralis*	0		0		0		1.2%	(0.1–4.3%)	0.2%	(0.0–0.9%)
*Trichuris* *trichiura*	0		0.2%	(0.0–1.0%)	2.1%	(0.2–7.3%)	1.2%	(0.1–4.3%)	0.6%	(0.2–1.4%)

## Discussion

This study estimated the prevalence of *T. solium* tapeworm infection in seven rural communities where PCVs serve in northern Peru. The overall prevalence of *Taenia* sp. egg-positivity by sedimentation and microscopy was 2.1%, which is considered hyper-endemic. [4] Prevalence as high as 4.3% by community was observed. These findings are higher in comparison to the results of many community-based studies that also estimated *Taenia* sp. tapeworm prevalence by microscopy, including studies performed in endemic areas of Peru [3], [4], [9] and other countries in Latin America [10]–[15] Africa [16]–[18] and northern Vietnam. [19] The overall prevalence of *T. solium* tapeworm infection was 1.5% with prevalence up to 2.9% by community. Similarly, these findings are higher when compared to the results of previous studies which report *T. solium* tapeworm prevalence by microscopy and PCR;[18]–[20] however, they are lower when compared to the results of studies that used more sensitive diagnostic methods such as coproantigen ELISA and EITB assay.[4], [16]–[18] Despite being high, the prevalence of *T. solium* tapeworm infection detected by our survey is likely an underestimation, as microscopy is poorly sensitive, missing 60–70% of *Taenia* sp. cases. [2] Nevertheless, our findings identified hyper-endemic communities and support our study hypothesis that a consortium of PCVs and university investigators can effectively perform large-scale, community-based public health research in remote regions of Peru.

To our knowledge, ours is the first study utilizing PCVs to facilitate local surveillance for *T. solium* tapeworm infection. This expansive stool survey illustrates the feasibility of using PCVs to identify study sites, assist with access and entre into those sites, recruit participants, and facilitate specimen collection, as well as highlights the utility of this concept, providing a template for future research studies. As demonstrated, PCVs can be a practical intermediate between researchers and rural communities. 90% of all PCVs have at least a bachelor’s degree, live as their communities do, and work on their behalf. [21], [22] All PCVs receive a minimum of two months of cultural, linguistic, and project-specific training, and approximately 20% receive additional specific training in health issues. [22] There are isolated published instances of PCVs working with outside organizations to offer treatment to their communities, such as a community eye health program in Samoa and a hernia repair clinic in Latin America. [23], [24] Peace Corps Volunteers commonly work in training programs, health education, and health promotion initiatives – one notable example is the HIV/AIDS programs in Africa.[25]–[27] The medical literature contains studies showing that PCVs are indirectly involved in health related issues; however, no published examples of PCVs facilitating public health research amongst their communities exists.

The prevalence of parasitic infection was higher in Piura than Cajamarca, where high endemicity of intestinal parasites and the presence of *Taenia* sp. have been previously reported.[28]–[30] The fact that we did not find *Taenia* sp. egg-positive stool samples in Cajamarca, where pigs are also raised freely, was surprising; however, there are several possible explanations. We did not formally investigate pig husbandry, yet we observed a greater number of free-roaming pigs in the Piura sites compared to the Cajamarca sites. Similarly, we did not investigate access to sanitary facilities; however, the most recent national census (2007) indicates that the communities we surveyed in Cajamarca have better access to sanitary facilities, specifically latrines, than the communities we surveyed in Piura. The presence of free-roaming pigs and the absence of latrines increase the risk for porcine cysticercosis [31]–[34] which has been found to be hyper-endemic in Piura. Jayashi et al. reported seroprevalence up to 45.6% in Piura, Morropon district. [35] Proximity to a cysticercosis-infected pig increases the risk for human *T. solium* tapeworm infection. [9] Participants were not randomly selected, and it is possible that those who participated in Piura were more likely to have intestinal parasites, including *Taenia* sp., than those who participated in Cajamarca. We collected and analyzed a single stool sample from each participant. Parasites shed eggs intermittently, thus the stool could have been produced at a time when the tapeworm was not shedding eggs. In such a case, infection would have gone undetected. Projects designed to control parasitic infection in humans and animals (primarily cattle) have been implemented in several districts in Cajamarca, including communities in the district of Baños del Inca. [36] Such projects could also explain the observed differences; to our knowledge, however, no such programs were in effect in the communities we surveyed. It should be mentioned that several groups have actively attempted to control *T. solium* in endemic areas by applying mass human chemotherapy. [13], [37], [38] A combined mass human and porcine chemotherapy trial was performed by the CWG in Peru in the Quilcas district, department of Junín, [39] and a longitudinal cysticercosis elimination program in Tumbes, located along Peru’s northern coast, is currently in effect.[40]–[44] However, no such programs have been implemented by the CWG in Peru in the sites we investigated in this study.

This study had a few key strengths. We obtained a large sample size, as PCVs secured high participation rates. We also recorded high prevalence of *T. solium* tapeworm infection in communities not previously surveyed. This study also had a few limitations, the most notable of which is the absence of application of a sensitive coproantigen ELISA for human taeniasis, [45], [46] a test that can detect 2 to 3 times as many confirmed cases of taeniasis as microscopy. [4] To that point, *Taenia* sp. and *T. solium* tapeworm prevalence are likely to be underestimated. Similarly, we were unable to identify the species of *Taenia* in 15 egg-positive participants. All 34 participants who were treated and expelled *Taenia* proglottids were infected with *T. solium,* thus we expect the 15 participants for whom the species is unknown to have *T. solium* as well. Yet, we cannot rule out the possibility that these participants were infected with *Taenia saginata (T. saginata)*, as this species of *Taenia* has been reported in northern Peru. It is important to note, however, that *T. saginata* is rare and has been found in only 10% of tapeworm carriers in the neighboring department of Tumbes (Garcia HH, unpublished data). [41].

## Conclusion

This study demonstrates that synergy between researchers and the Peace Corps can be an effective means to conduct large-scale, community-based studies in remote areas. In our case, collaboration allowed for the discovery of hyper-endemic communities of *T. solium* tapeworm infection, and connected doctors, epidemiologists, and scientists to those communities through PCVs in a mutually beneficial manner. Peace Corps Peru and the CWG in Peru have begun discussions on subsequent projects, the development of appropriate, community-specific interventions, as well as the incorporation of basic epidemiologic principles and taeniasis/cysticercosis education into the PCV training continuum.
